# Dynamic Compression Plating Versus Antegrade Intramedullary Nailing for the Treatment of OTA/AO 12-A Fractures: A Retrospective Cohort Study

**DOI:** 10.7759/cureus.52472

**Published:** 2024-01-17

**Authors:** Bander S Alrashedan, Mohammed M Almalki, Norah I Alromaih, Bashah Almustanir, Hussain M Alyassain, Bandar Sahli

**Affiliations:** 1 Orthopedic Surgery, King Saud Medical City, Riyadh, SAU; 2 Orthopedics, King Saud Medical City, College of Medicine, Riyadh, SAU

**Keywords:** clinical outcomes, cost-effectiveness, dynamic compression plating, intramedullary nailing, humerus diaphyseal fractures

## Abstract

Background: Fractures of the humerus diaphysis are common and often result from motor vehicle accidents (MVAs). Treatment methods range from nonoperative approaches to various operative techniques, including antegrade intramedullary nailing (AIMN) and dynamic compression plate (DCP) fixation. This study aimed to compare the cost effectiveness and outcomes of plating and nailing for humerus diaphyseal fractures.

Methods: A retrospective cohort study involving 59 cases of humerus diaphyseal OTA/AO 12-A fractures was conducted at King Saud Medical City (KSMC), a level I trauma center located in the center region in Riyadh, Saudi Arabia. Patients treated with AIMN, anterolateral plating, or posterior plating were included. Data on demographics, clinical parameters, radiographic healing, and costs were collected and analyzed.

Results: The average surgical duration was shorter in the AIMN group compared to the anterolateral and posterior plating groups but with no statistical significance (P > 0.05). The average length of stay (LOS) was shorter, and the change in hemoglobin levels was lower in the AIMN group when compared to other groups but without a statistically significant difference (P > 0.05). The average cost of AIMN was significantly higher than that of anterolateral and posterior plating groups (P < 0.0001).

Conclusion: While both nailing and plating procedures are options for treating OTA/AO 12-A fractures, AIMN carries a higher overall procedural cost. The practice of drain placement in our study population is likely the cause of the increased LOS in the plating groups. Relative additional analgesic requirements were associated with AIMN. Surgeons should consider meticulous hemostasis to avoid drain placement, which can decrease LOS, thus possibly decreasing unnecessary treatment costs of humerus shaft fractures.

## Introduction

Fractures of the humerus diaphysis account for 1-3% of all fractures, and they have been reported to be more common in males, with a peak incidence in the third decade [1]. The most common cause of these fractures is motor vehicle accidents (MVAs) [1]. These fractures can be managed by operative and nonoperative methods. Most of these injuries are treated nonoperatively [2]. The advantages of nonoperative treatment options for these fractures by functional braces or casts preceded by a short period of traction have been highlighted by many authors [3-5]. Although nonoperative methods commonly give excellent results, some issues may persist, which has promoted the development of different internal and external fixation techniques [6]. Several studies have demonstrated that simple humerus shaft fractures, such as AO/OTA 12-A, have an increased risk of nonunion because of the deforming force on the fracture edges [7-9]. Conservative management for diaphyseal humerus fractures has high rates of union and is the gold standard despite the increased trend toward surgical treatment, likely due to the higher union rates and the ability to achieve and maintain anatomic fracture reduction [10]. Choosing the better option between antegrade intramedullary nailing (AIMN) and dynamic compression plate (DCP) for fixing humerus diaphysis fractures is still controversial. These implants have been reported to have advantages and disadvantages varying from one study to another [2,11-15].

For instance, a study found that plating is a better option for diaphyseal humerus fractures due to the decreased need for secondary procedures and better post-operative shoulder function [2]. Another study similarly concluded that nailing has a negative effect on shoulder function with increased risk of iatrogenic fracture comminution [11]. Newer-generation straight IMN, however, has shown better shoulder function and fracture healing rate when compared to previous generation curved nails [12]. Another study showed that functional outcomes were similar when the two procedures were compared; however, there was a higher overall complication rate with nailing [15].

In this study, we aim to examine the differences between AIMN and anterolateral and posterior plating in terms of surgical duration, length of stay (LOS), need for additional narcotics, change in hemoglobin, heart rate, and cost.

## Materials and methods

Study design

This is a retrospective cohort study conducted at King Saud Medical City (KSMC), which is a level I trauma center located in the center region in Riyadh, Saudi Arabia. It is considered the largest Ministry of Health community trauma center and a destination for referral of secondary hospitals. We included cases presented to our hospital from January 1, 2015, to December 20, 2021, with a diagnosis of humerus shaft fractures after obtaining approval from the Institutional Review Board of KSMC (reference: H1RI-31-Aug23-02), who are responsible for all experimental protocols and carry the approval when appropriate. The primary outcome measure is to assess the differences between AIMN and anterolateral plating and posterior plating for the treatment of diaphyseal fractures of the humerus. The secondary outcome measure is to assess the differences between AIMN and all plating procedures for the treatment of diaphyseal fractures of the humerus.

Patient selection

Included samples were cases that presented acutely with an isolated diaphyseal transverse or short oblique humerus fracture (AO/OTA 12A) acutely and managed with either AIMN (Group 1), anterolateral plating (Group 2), or posterior plating (Group 3) above the age of 14 years old. We excluded cases that were associated with other injuries, pathological fractures, or comminuted fractures or proximal or distal metaphyseal in the location or fracture non-union. A follow-up was necessary to assess radiographic healing; all cases were followed for at least two years. There were 266 cases found in medical records; 59 cases met our inclusion criteria, and 207 cases were excluded due to either associated multiple injuries operated along the humerus in the same sitting, which affected the surgical duration, or fractures involving the proximal or distal humerus.

Surgical procedures and data collection

**Figure 1:**
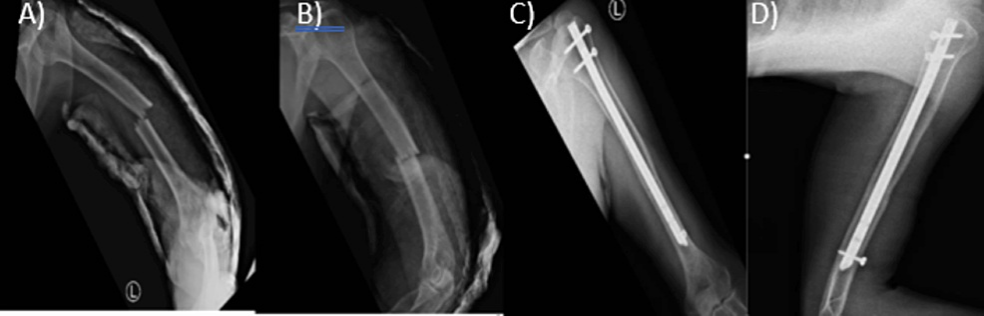
Anteroposterior (AP) and lateral radiographs of the left humerus shaft fracture fixed with an antegrade intramedullary nail (AIMN). (A) Preoperative anteroposterior (AP) view, (B) preoperative lateral view, (C) postoperative antegrade intramedullary nail (AIMN) fixation AP view, (D) postoperative AIMN fixation lateral view

**Figure 2:**
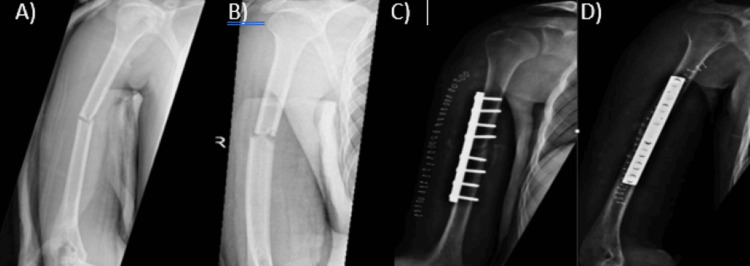
Anteroposterior (AP) and lateral radiographs of the right humerus shaft fracture fixed with an anterolateral plate. (A) Preoperative anteroposterior (AP) view, (B) preoperative lateral view, (C) postoperative anterolateral plate fixation AP view, (D) postoperative anterolateral plate fixation lateral view

**Figure 3:**
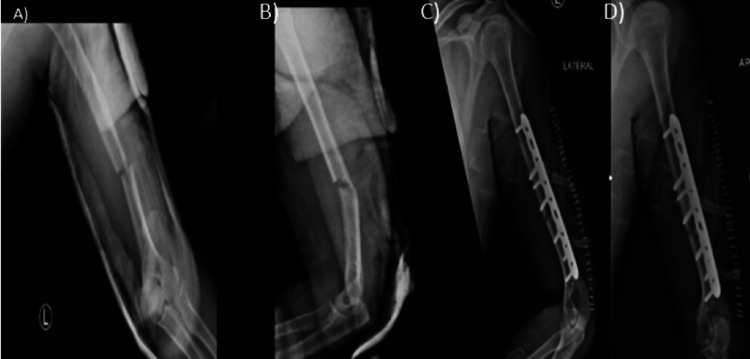
Anteroposterior (AP) and lateral radiographs of the left humerus shaft fracture fixed with a posterior plate. (A) Preoperative anteroposterior (AP) view, (B) preoperative lateral view, (C) postoperative posterior plate fixation AP view, (D) postoperative posterior plate fixation lateral view

AIMN cases were done using the Expert Humeral Nailing System developed by DePuy Synthes (Indiana, USA) (Figure 1), while plating procedures were done using narrow 4.5 mm limited contact dynamic compression plates either with an anterolateral approach (Figure 2) or posterior approach (Figure 3) with the use of conventional screws. All cases were done or supervised by certified orthopedic consultants at our institute. All surgeons who operated on the study population were found to have done at least one case in each treatment group. The immediate postoperative protocol consisted of a fully active and passive range of motion of the shoulder and elbow with restricted weightlifting for three weeks, followed by gradual resumption of daily living activity until reaching the baseline functional status. The usual postoperative pain regimen consisted of an intravenous 50 mg of tramadol regularly given three times a day and an intravenous 1 gram of paracetamol regularly given four times a day while in the hospital. Rescue narcotics used was morphine, which was prescribed if the regular pain medications failed to improve pain in our study population. Demographic data included age, gender, and smoking status. Clinical data included were surgical duration, which was obtained from the time-out and sign-off sheets, LOS, change of hemoglobin level from preoperative to postoperative workup, change in the average heart rate from preoperatively to postoperatively, required surgical procedure for the patient, postoperative neurological status, and need for an additional opioid dose at the day of the procedure. Procedure costs were obtained from the Health Economics Department in US dollar. The specific surgical code of a one-day stay of plating (anterior or posterior) is $1,110.67, while AIMN costs $2,586.67. An additional one stay at the hospital costs $186.67 regardless of the procedure. Radiographic data included fracture geometry, location, and healing on follow-up. Fracture union is defined as the complete formation of bone at the fracture site with no radiolucent lines on follow-up radiographs.

Statistical analysis

All cases were managed and followed at our hospital. Data were collected in an Excel sheet and were analyzed using IBM SPSS Statistics for Windows, version 21 (released 2012; IBM Corp., Armonk, New York, United States). Patients were stratified into three groups: AIMN group, anterolateral plating group, and posterior plating group. Analysis of the demographic data including age, smoking, and gender was done between the groups to see any differences that could affect the results when compared for the primary outcome. A comparison between the three groups was made in terms of the average surgical duration, average LOS, average change in heart rate and hemoglobin levels, cost, and need for additional narcotic use. The same comparison was made between AIMN (Group 1) and plating procedures (Group 2, Group 3) to assess the overall differences between the nailing and plating procedures. Categorical values were analyzed using a chi-square test. Numerical values were analyzed using Student's t-test when two groups were compared and analysis of variance (ANOVA) when multiple groups were compared. Data normality was assessed using Shapiro-Wilk test. A P value of less than 0.05 is considered statistically significant.

## Results

Patients' characteristics and demographics

The study included 59 cases, 21 (35.59%) AIMN, 24 (40.67%) anterolateral plating, and 14 (23.72%) posterior plating. All cases demonstrated a fracture union within 16 weeks. There were 11 (18.64%) preoperative radial nerve palsy, which all recovered within three months postoperatively; three (27.27%) of those were managed with AIMN, four (36.36%) were managed by anterolateral plating, and five (45.45%) of the cases were managed with posterior plating. Of all the cases, 55 (93.22%) were male and four (6.78%) were female patients. There were no cases of iatrogenic postoperative neurological injury, and all cases showed radiographic union on follow-up. Upon comparing the surgical treatment groups, there were no statistically significant differences in terms of average age, male-to-female ratio, and smoking status (P > 0.05) (Table 1).

**Table 1:** Patient characteristics and demographics The data are presented in the form of mean±SD.

P value	Smoking status (smoker vs. non-smoker)	P value	Gender (M:F)	P value	Age	Groups
0.234	4:21	0.45	18:3	0.076	39.7 ± 16.44	Nailing (21)
	1:23		23:1		29.96 ± 9.22	Anterolateral plating (24)
	1:13		14:2		28.43 ± 8.97	Posterior Plating (14)

Outcome of nailing vs. anterolateral and posterior plating

The average surgical duration was found to be 122 ± 33.78 minutes in the AIMN group, while it was found to be 142 ± 64.87 minutes and 152 ± 66.22 minutes in the anterolateral plating and posterior plating groups, respectively, with no statistically significant significance (P > 0.05). The average LOS was 1.9 ± 0.94 days in the AIMN compared to 3.29 ± 2.79 days and 4.79 ± 8.8 days in the anterolateral plating and posterior plating groups, respectively, with no statistically significant difference (P > 0.05). The average change in the hemoglobin level was found to be 1.14 ± 1.19 in the AIMN group, while it was 1.83 ± 2.87 and 2.1 ± 1.39 in the anterolateral and posterior plating groups, respectively, with no statistically significant significance (P > 0.05). The average change in heart rate was 3.14 ± 2.37 beats per minute in the AIMN compared to 4.08 ± 3.22 and 4.43 ± 2.06 beats per minute in the anterolateral and posterior plating groups, respectively, with no statistically significant significance (P > 0.05). Additional midnight opioid dose was required in eight (38.1%) patients of the AIMN group compared to eight (33.33%) and six (42.86%) patients of the anterolateral and posterior plating groups, respectively, with no statistically significant significance (P > 0.05). The average cost of the nailing procedure was found to be significantly higher than that of the anterolateral plating and posterior plating procedures (P < 0.0001), with the average cost of anterolateral to be $1,153.78 ± 369.75 compared to AIMN (mean $2,568.11 ± 16.17) and posterior plating (mean: $1,322.68 ± 74.64) (Table 2).

**Table 2:** Clinical data and outcome comparing AIMN to anterolateral and posterior plating AIMN: antegrade intramedullary nailing, HGB: hemoglobin The data are presented in the form of mean±SD.

P value	Procedure		Parameter
0.266	122 ± 33.78	Nailing	Average surgical duration
	142 ± 64.87	Anterolateral plating	
	152 ± 66.22	Posterior plating	
0.2	1.9 ± 0.94	Nailing	Average length of stay
	3.29 ± 2.79	Anterolateral plating	
	4.79 ± 8.8	Posterior plating	
0.356	1.14 ± 1.19	Nailing	Average change in HGB
	1.83 ± 2.87	Anterolateral plating	
	2.1 ± 1.39	Posterior plating	
0.33	3.14 ± 2.37	Nailing	Average change in heart rate
	4.08 ± 3.22	Anterolateral plating	
	4.43 ± 2.06	Posterior plating	
0.839	N=8 (38.1%)	Nailing	Required additional narcotics
	N=8 (33.33%)	Anterolateral plating	
	N= 6 (42.86%)	Posterior plating	
<0.0001	$2568.11 ± 16.17	Nailing	Average cost

Outcome of nailing vs. plating

When the AIMN group (n = 21, 35.60%) was compared to the plating group (n = 38, 64.40%), it was found that the average surgical duration was lower than in cases who underwent AIMN compared to the plating but without a statistical significance (P > 0.05). No statistically significant difference was found when we compared the average LOS between the antegrade and plating group with a longer hospital stay in the plating group (P > 0.05). The average change in hemoglobin level and heart rate from before and after the surgery was lower in the AIMN group than in the plating group but without statistical significance (P > 0.05). Additional narcotic use was found in 38.1% (n = 8) of the AIMN group compared to 36.84% (n = 14) of the plating group, with a statistically significant difference (P < 0.05). The average cost of AIMN was found to be significantly higher than the average costs of plating procedures (mean: $2,568.11 ± 176.10 vs. mean: $1,187.57 ± 74.59, respectively) (P < 0.0001) (Table 3).

**Table 3:** Clinical data and outcome comparing AIMN to plating AIMN: antegrade intramedullary nailing, HGB: hemoglobin The data are presented in the form of mean±SD.

P value	Procedure		Parameter
0.067	122 ± 33.78	Nailing	Average surgical duration
	142 ± 64.87	Plating	
0.13	1.9 ± 0.94	Nailing	Average length of stay
	3.84 ± 5.71	Plating	
0.164	1.14 ± 1.19	Nailing	Average change in HGB
	1.93 ± 2.41	Plating	
0.147	3.14 ± 2.37	Nailing	Average change in heart rate
	4.21 ± 2.82	Plating	
0.009	N=8 (38.1%)	Nailing	Required additional narcotics
	N=14 (36.84%)	Plating	
<0.0001	$2568.11 ± 176.10)	Nailing	Average cost
	$1187.57 ± 74.59	Plating	

## Discussion

Conservative management of diaphyseal humerus shaft fractures is considered the gold standard treatment option despite the lower overall union rates reported in the literature, which range from 77% to 100%, compared to plating, which was reported to range from 87% to 96% [10]. The union was shorter in the absolute stability fixation of simple diaphyseal humerus shaft fractures compared to relative stability fixation [14]. Conservative management in our institute does not commonly occur, especially in transverse or short oblique fractures where they have a high tendency to displace and shorten, and for this reason, the two surgical options were included in this study.

Numerous randomized controlled trials and systematic reviews have examined plate fixation compared to AIMN fixation for humeral diaphyseal fractures. Despite these efforts, no definitive conclusion has emerged regarding the superiority of one fixation method over the other when it comes to operative time, time to union, and duration of hospital stay. While nailing might be favored in pathological fractures or in cases where there is extensive comminution or associated severe soft tissue injury, better functional outcomes and lower complications were in favor of plating in most literature [2,11-15]. In our study, a key finding highlights the significantly higher cost associated with AIMN fixation compared to plating for the treatment of humeral diaphyseal fractures. Notably, the AIMN fixation also demonstrated an increased requirement for midnight narcotic medication, whereas no significant differences were observed between the two procedures in terms of operative time, time to union, intraoperative blood loss, and hospital LOS. The overall prevalence of radial nerve injury following a diaphyseal humerus fracture was 11.8% [16]. In our study, the rate of radial nerve palsy was considerably higher (18.64%); the only possible explanation is the significant rate of high mechanism injury cases presenting to our hospital, which is known to be a risk factor for radial nerve palsy.

Over the past decades, the desire for cost savings in orthopedic surgery has grown. Several studies have compared the costs of surgical techniques and fixation types for various orthopedic procedures. For example, Wasterlain et al. underscored the pivotal role of cost for surgeons when faced with two procedures yielding equivalent outcomes [17]. Previous research has also found that physicians' understanding of cost differences for humeral diaphyseal fractures and other fractures significantly impacts surgical technique selection and implants used [17]. Our study aligns with these trends, revealing that open reduction and internal fixation with plating is associated with lower costs than AIMN, despite a slightly prolonged postoperative stay in the plating group. Notably, each day of hospital stay incurs a cost of $186.67. The enhanced cost of AIMN is found in the pre-made surgical code of the procedure, and lower implant costs could probably decrease the significant difference between the two treatment options. Although the LOS was statistically insignificant, it is worth noting that the plating group exhibited a longer hospital stay (3.84 days) compared to the AIMN group (1.89 days), consequently leading to higher costs and placing an additional burden on the hospital. This finding corroborates prior literature, such as the study by Den Hartog et al., which reported an average LOS of three days for the plating group compared to two days for the AIMN group [18]. However, it is essential to acknowledge that these findings were not statistically significant, given the substantially higher cost of AIMN ($2,398) in contrast to plating ($533).

We found a greater use of midnight rescue narcotic medication in the AIMN group compared to the plating group. This disparity may be attributed to the reaming process and the disruption of endosteal blood supply, leading to increased intraosseous pressure [19]. Biomechanically, AIMN introduces a disruption in the endosteal blood supply, which accounts for approximately 80% of bone vascularization. This disruption triggers a hyperemic reaction and elevated inflammatory markers, ultimately contributing to increased pain perception [19,20]. We could not find any evidence of pain level differences when comparing plating and nailing. However, reports have shown that open treatment of intertrochanteric femur fractures is more painful than proximal femur nailing; it was attributed to the dissection of hip abductors during dynamic compression plating placement [21,22]. Open treatment of humerus shaft fracture involves dissection through the intermuscular plane that is away from any muscular insertion or origin, thus possibly explaining the less additional narcotic use in the plating group compared to nailing where the rotator cuff tendon is opened and repaired during the surgical procedure.

All fractures assessed in our study exhibited complete union within a maximum of 16 weeks, with no instances of revision within a two-year follow-up period for both fixation groups. These results mirror existing literature. For instance, Den Hartog et al. [18] reported no statistically significant difference in union rates between plating and nailing. Moreover, systematic reviews and meta-analyses conducted by Beeres et al. [23] and Wen et al. [24] demonstrated no statistically significant difference in union rates (P > 0.05). Beeres et al. [23] also highlighted a statistically significant difference in time to union, with an average of 12 weeks for nailing and 14 weeks for plating [23]. This was justified by the minimally invasive techniques in AIMN, which preserve the blood supply to the periosteum, unlike open reduction with internal fixation (ORIF), which carries a risk of serious destruction of the periosteum humeral blood supply. All the fractures included in this study were either transverse or short oblique fractures (AO/OTA 12A), predisposing them to high nonunion risk. Nonetheless, all of them displayed complete union, leading us to consider that the fixation technique holds greater significance than the choice of implant type [7-9].

Several studies focusing on the operative duration of both procedures were done with variable and conflicting results. Our findings were consistent with the results of Wen et al. [24], who concluded that there was no difference in operative duration between the two procedures. On the other hand, several other studies concluded that operative duration was statistically significant and shorter in the nailing group compared to the plating group [18,23-26]. These conflicting results are due to several reasons, mainly that humeral nailing was done by different surgeons in our institution. Some reports found that operative blood loss was significantly different between nailing and plating, in favor of less blood loss in the nailing group [24,26]. This differs from the findings in this study, where there was no significant difference in blood loss; there was an increased drop in hemoglobin in the ORIF group compared to the AIMN group, but without a statistically significant difference (P > 0.05). It is important to note that our study examined postoperative hemoglobin change, unlike the cited studies, which examined intraoperative blood loss. We hypothesize that blood loss could be related to the increased amount of hidden blood loss (third-space blood loss) phenomena related to the reaming process. For example, Wang et al. [27] examined that the hidden blood loss in tibia fractures was higher in the AIMN group than in the ORIF group. This is also supported by Minhas et al. [28], who concluded that there is an increased postoperative transfusion requirement in AIMN fixation compared to plate fixation in extra-articular tibia fractures.

## Conclusions

Nailing and plating procedures are valid options for treating acutely presented AO/OTA 12A fractures. In this study, we found higher overall costs in nailing procedures despite the shorter LOS at our institute, while fracture healing, change in hemoglobin, and heart rate were not significantly different between the two procedures. AIMN can still be favored in pathological fractures or in cases with an associated soft injury. The practice of drain placement in the plating group could have potentially increased the overall LOS compared to nailing procedures. Therefore, surgeons may rely on meticulous hemostasis to avoid unnecessary longer hospital stays without using drains after plating of diaphyseal humerus shaft fractures. Moreover, the reason behind the non-significant difference in the LOS is the fact that additional narcotic requirements were more in the nailing group. Cost implications can be substantial, and more studies could potentially delineate the indications of an implant or possibly the development of lower-cost intramedullary nails. These findings can help surgeons and healthcare providers make informed decisions regarding surgical technique selection for humeral diaphyseal fractures.
